# Identification of quantitative trait loci governing early germination and seedling vigor traits related to weed competitive ability in rice

**DOI:** 10.1007/s10681-020-02694-8

**Published:** 2020-09-19

**Authors:** Niña Gracel B. Dimaano, Jauhar Ali, Anumalla Mahender, Pompe C. Sta. Cruz, Aurora M. Baltazar, Maria Genaleen Q. Diaz, Yun Long Pang, Bart L. Acero, Zhikang Li

**Affiliations:** 1 University of the Philippines Los Baños, 4031 Los Baños, Laguna Philippines; 2 Rice Breeding Platform, International Rice Research Institute (IRRI), 4031 Los Baños, Laguna Philippines; 3 State Key Laboratory of Crop Biology, College of Agronomy, Shandong Agricultural University, Taian, 271018 People’s Republic of China; 4 National Key Facility for Crop Gene Resources and Genetic Improvement, Institute of Crop Science, Chinese Academy of Agricultural Sciences (CAAS), Beijing, 100081 People’s Republic of China

**Keywords:** Early seed germination, Early seedling vigor, Selective introgression lines, Quantitative trait loci, Single nucleotide polymorphism, Weed competitive ability

## Abstract

Weed competitive ability (WCA) is vital for the improvement of grain yield under direct-seeded and aerobic rice ecosystems where weeds are a major limiting factor. Early seed germination (ESG) and early seedling vigor (ESV) are the crucial traits for WCA. This study attempted to map the quantitative trait loci (QTLs) and hotspot regions governing ESG and ESV traits. A total of 167 BC_1_F_5_ selective introgression lines developed from an early backcross population involving Weed Tolerant Rice 1 (WTR-1) as the recipient parent and Y-134 as the donor parent were phenotyped for ESG and ESV traits. Analysis of variance revealed significant differences in ESG-related traits except for root length and in ESV-related traits except for plant height at 7 days after sowing. A total of 677-high quality single nucleotide polymorphism (SNP) markers were used to analyze the marker-trait association from a 6 K SNP genotyping array. Forty-three QTLs were identified on all chromosomes, except on chromosomes 4 and 8. Thirty QTLs were contributed by a desirable allele from Y-134, whereas 13 QTLs were from WTR-1. Twenty-eight of the identified genetic loci associated with ESG and ESV traits were novel. Two QTL hotspot regions were mapped on chromosomes 11 and 12. The genomic regions of QTL hotspots were fine-tuned and a total of 13 putative candidate genes were discovered on chromosomes 11 and 12 collectively. The mapped QTLs will be useful in advancing the marker aided-selection schemes and breeding programs for the development of rice cultivars with WCA traits.

**Electronic supplementary material:**

The online version of this article (10.1007/s10681-020-02694-8) contains supplementary material, which is available to authorized users.

## Introduction

Rice is a major food crop for half of the world’s population. By 2050, 42% more rice yield will be needed to meet the rapidly growing global demand (Ray et al. 2013). Unfortunately, the increase of global rice production is constrained by various biotic and abiotic stresses in diverse rice ecosystems (Pandey et al. 2017). The future threat to natural resources, rising labor shortages, declining arable lands, increasing prices of fertilizer and pesticide inputs, energy scarcity, and changing climatic conditions are the major factors contributing to the decrease in rice production (Singh et al. 2013). To overcome these constraints, shifting from the conventional puddled transplanted rice system to direct-seeded rice (DSR) is the most promising strategy (Chauhan and Abugho 2013; Mahender et al. 2015). DSR has several advantages such as reducing water usage by 35% to 75%, decreasing labor demand, shortening the crop duration, mitigating methane gas emissions, and lowering the cost of cultivation (Mahender et al. 2015). However, the vigorous growth of weeds is one of the major biological constraints to attain optimal grain yield in the DSR system (Chauhan et al. 2015).

Many options exist to control weeds in the DSR system, such as tillage operations and herbicide applications. However, tillage approaches are laborious, while herbicide use requires multiple applications during the cropping season, thus increasing the financial burden on farmers (Rahman et al. 2012). Appleby et al. (2002) have estimated that more than USD 100 billion are lost annually due to weed-control practices globally. Therefore, urgent attention is required to develop alternative sustainable weed management technologies for the DSR system. Breeding for cultivars with weed competitive ability (WCA) is a promising strategy to reduce tillage operations and herbicide inputs in the DSR system. Rice cultivars with WCA can suppress the growth of weeds without a yield penalty under weedy conditions (Dimaano et al. 2017). WCA is a complex and polygenic trait, which is governed by several agro-morphological features related to early seed germination (ESG) and early seedling vigor (ESV). WCA is significantly involved in DSR and aerobic rice cultivation (Okami et al. 2011; Mahender et al. 2015).

In the DSR system, there is no standing water and seedling size advantage to suppress weed growth and emergence. The ESG and ESV traits are crucial in the early crop establishment and successful competition of rice cultivars against weeds (Haque et al. 2007; Mahajan et al. 2014; Dimaano et al. 2017). In order to identify the weed competitive rice cultivars for the DSR system, the rice plant must have early germination capacity and faster seedling vigor. ESV is a highly repeatable trait that can be used to discriminate rice cultivars that have a strong or weak ability for weed competition (Caton et al. 2003). Several researchers reported that germination rate and seedling vigor had significant positive correlations with field emergence, seedling establishment, germination rate, plant height, and seedling dry weight (Yang et al. 2010; Mahajan et al. 2014; Dimaano et al. 2017; Zhang et al. 2017). The rate of seedling emergence, ability to germinate, and early seedling growth of shoot and root traits are the major factors for crop establishment, which provides superior root growth that can help in the absorption of more nutrients (Matsushima and Sakagami 2013; Singh et al. 2015; Khan et al. 2016). The uniformity of seedling growth and germination percentages were significantly associated with strong, vigorous crop growth and better seedling establishment, which can influence the improvement of yield (Cui et al. 2002a; Diwan et al. 2013; Dang et al. 2014). In addition, several traits that are significantly linked to WCA include plant height (Mahajan et al. 2014), tiller number (Kaur and Singh 2014), leaf area index (Rao et al. 2007), mesocotyl elongation (Lee et al. 2012), early crop biomass (Ni et al. 2000), shoot and root dry weight (Zhao et al. 2006a, b; Lu et al. 2007; Yang et al. 2010; Okami et al. 2011), and canopy ground cover (Anwar et al. 2012).

The identification of quantitative trait loci (QTLs) for ESG and ESV traits which are fundamental for WCA will provide useful information in the marker-aided selection for weed competitive genotypes. Therefore, this study was conducted to investigate the QTLs and hotspot regions governing the ESG and ESV traits using a BC_1_F_5_ early backcross selective introgression lines (SILs) derived from a cross between Weed Tolerant Rice-1 (recipient parent) and Y-134 (donor parent). In this study, we assessed the phenotypic variance of the key traits related to ESG and ESV and mapped the QTLs governing these traits using a high-quality single nucleotide polymorphism (SNP) array. Our results revealed novel QTLs for ESG and ESV traits, thus advance the understanding of the association of rice genomic regions and key WCA traits.

## Materials and methods

### Plant materials

A total of 167 BC_1_F_5_ generation of early backcross SILs of a Green Super Rice (GSR) IR2-6 population were derived from a cross between Weed Tolerant Rice 1 (WTR-1) as a recipient parent and Y-134 as a donor parent developed at the International Rice Research Institute (IRRI). WTR-1 is the GSR recipient parent from South China, and is a widely adaptable rice variety with WCA traits; while Y-134 is a high-yielding variety and is a potential donor for agronomic traits which were used in the GSR-breeding program (Dimaano et al. 2017; Pang et al. 2017; Ali et al. 2018). Junglerice [*Echinochloa colona* (L.) Link], one of the most dominant grass weed species in rice fields, was used in the pot experiment to simulate weed competition.

### Phenotyping of early seed germination (ESG) traits

Seed dormancy of SILs was broken by incubating the seeds at 50 °C for 4 days (Jennings and de Jesus 1964). Two replications of 25 seeds from each of the 167 SILs were placed randomly in a 9-cm-diameter Petri dish lined with two layers of wet filter paper and kept in a germination chamber set at 30 °C 12 h-photoperiod for 14 days. Seeds showing a 2 mm radicle length were considered germinated. Seed germination was counted at two different intervals: 48 h after seed placement as first germination count (1st GC) and 7 days after seed placement as second germination count (2nd GC). Germination percentage (GP-1) was determined as the ratio of 1st GC to 2nd GC. At 14 days after seed placement, data were collected for shoot length (SL), root length (RL-1), total fresh weight of germinated seeds (TFGS), and average fresh weight (AFW) of germinated seeds. The AFW was computed by dividing TFGS to the total number of seeds that germinated. SL was measured from the collar region to the tip of the topmost leaf. RL-1 was measured from the collar region down to the tip of the longest root. The total dry weight of germinated seeds (TDGS) was measured after drying at 70 °C for 5 days. The average dry weight (ADW) was computed by dividing TDGS by the total number of seeds that germinated. Vigor index (VI-1) was calculated by multiplying GP-1 by TDGS (Table 1).

**Table 1:** Rice agro-morphological characteristics investigated for early seed germination (ESG) and early seedling vigor (ESV) traits

No.		Trait observations	Description of trait
*Early seed germination (ESG) traits*			
1	1st GC	1st germination count	Number of germinated seeds after 48 h
2	2nd GC	2nd germination count	Number of germinated seeds after 7 days
3	GP-1	Germination percentage	The ratio of the 1st germination count to the 2nd germination count
4	SL	Shoot length (cm)	Measured from the collar region to the tip of topmost leaf
5	RL-1	Root length (cm)	Measured from collar region down to the tip of the longest root
6	TFGS	Total fresh weight of germinated seeds	Total fresh weight of all seeds that germinated
7	TDGS	The total dry weight of germinated seeds	The total dry weight of all seeds that germinated after drying at 70 °C for 5 days
8	AFW	Average fresh weight	TGFS/number of seeds that germinated
9	ADW	Average dry weight	TDGS/number of seeds that germinated
10	VI-1	Vigor index	Germination percentage multiplied by the total dry weight of germinated seeds
*Early seedling vigor (ESV) traits*			
1	GC	Germination count	Number of seeds that germinated
2	GP-2	Germination percentage	The ratio of the germinated seeds to the total number of seeds
3	PH at 7 DAS	Seedling plant height (cm) at 7 DAS	Plant height at 7 days after sowing
4	PH at 14 DAS	Seedling plant height (cm) at 14 DAS	Plant height at 14 days after sowing
5	PH at 21 DAS	Seedling plant height (cm) at 21 DAS	Plant height at 21 days after sowing
6	PH at 28 DAS	Seedling plant height (cm) at 28 DAS	Plant height at 28 days after sowing
7	NL at 7 DAS	Number of leaves at 7 DAS	Number of leaves at 7 days after sowing
8	NL at 14 DAS	Number of leaves at 14 DAS	Number of leaves at 14 days after sowing
9	NL at 21 DAS	Number of leaves at 21 DAS	Number of leaves at 21 days after sowing
10	NL at 28 DAS	Number of leaves at 28 DAS	Number of leaves at 28 days after sowing
11	NT	Number of tillers	Number of tillers per plant
12	LCC	Leaf chlorophyll content	Reading-based on Soil–Plant Analyses Development meter
13	VI-2	Vigor index	Germination percentage multiplied by total dry weight
14	LFW	Leaf fresh weight (g)	Fresh weight of leaves
15	LDW	Leaf dry weight (g)	The dry weight of leaves after drying at 70 °C for 5 days
16	RFW	Root fresh weight (g)	Fresh weight of roots
17	RDW	Root dry weight (g)	The dry weight of roots after drying at 70 °C for 5 days
18	TFW	Total fresh weight (g)	Measured by computing leaf fresh weight + root fresh weight
19	TDW	Total dry weight (g)	Measured by computing leaf dry weight + root dry weight
20	RL-2	Root length (cm)	Measured from collar region down to the tip of the longest root

### Phenotyping of early seedling vigor (ESV) traits

Two replications of 5 seeds of 167 SILs along with parents WTR-1 and Y-134 were sown in plastic pots filled with soil and grown until 28 days after sowing (DAS) for investigation of seedling vigor traits. The soil type used was Maahas clay loam (iso-hyperthermic mixed Typic-Tropudalf). A compound N-P-K fertilizer (14:14:14) was added to each pot based on field recommendations. One hundred seeds of jungle rice [*Echinochloa colona* (L.) Link] were randomly sown in each pot simultaneously with rice seeds to simulate weed competition. The pots were watered once daily, and the moisture was kept at direct-seeded non-flooded condition. At seven DAS, the germination count (GC) and germination percentage (GP-2) was measured for each SIL. GP-2 was computed by dividing GC by the total number of initial seeds. Seedling plant height (PH) and the number of leaves (NL) were measured at 7, 14, 21, and 28 DAS. The number of tillers (NT) and leaf chlorophyll content (LCC) was recorded at 28 DAS. After each observation at 28 DAS, the seedlings were uprooted carefully, shoots and roots were separated, and values for leaf fresh weight (LFW), root length (RL-2), root fresh weight (RFW), and total fresh weight (TFW) were collected. The plant samples were oven-dried at 70 °C for 5 days then measured for leaf dry weight (LDW), root dry weight (RDW), and total dry weight (TDW). Vigor index (VI-2) was computed by multiplying GP-2 by TDW. The weed density (WD), weed fresh weight (WFW), and weed dry weight (WDW) were collected at 28 DAS to correlate with rice seedling vigor performance and to assess WCA. Junglerice shoots were cut at the soil surface, weighed for WFW, oven-dried at 70 °C for 5 days, and weighed for WDW (Table 1).

### Statistical analysis

All phenotypic data of ESG- and ESV-related traits were analyzed using Plant Breeding Tools version 1.4 (IRRI 2017) for descriptive statistics including mean, minimum and maximum values, standard deviation, coefficient of variation (%), and correlation analysis and analysis of variance (ANOVA) at 1% level of significance. The heat maps of Pearson’s correlation coefficient were generated using the *corrplot* package and the broad-sense heritability was calculated based on the replicated data and random-effects ANOVA using the *lmer* package in R software (R Development Core Team 2012).

### DNA extraction and genotyping

Leaf samples were collected from each of the SILs and parents at 21 DAS. The genomic DNA was extracted and purified following a modified CTAB method (Murray and Thompson 1980) and quantified by using NanoDrop 8000 spectrophotometer (Thermo Scientific, USA). The concentration of the extracted DNA sample was adjusted to 50 ng μl^−1^ and used in the 6 K SNP array. DNA quantification, incubation, hybridization of bead chip, staining, and image scanning were performed according to the manufacturer’s instructions for the Illumina Infinium assay at the Genotyping Services Laboratory of IRRI. The resulting intensity data were processed for SNP calling by using genotyping module V2011.1 of Genome Studio software (Illumina Inc., San Diego, CA, USA). The genotypic data from the 6 K SNP array were filtered according to the methods described by Najeeb et al. (2020), and the obtained polymorphic SNPs between the parents were used to map the QTLs for ESG and ESV traits.

### QTL mapping

For the QTL analysis, the mean phenotypic trait values (across replications) of 167 SILs and the corresponding SNP marker data were used. A total of 677 polymorphic SNP markers covering > 90% of all chromosomes and their physical positions were used to construct the physical map (Table 2). The QTL mapping was performed by single-marker regression analysis using the function of single marker analysis (SMA) in IciMapping software v4.1 (www.isbreeding.net/software/?type=detail&id=18) (Meng et al. 2015). The threshold (−log *p*(F) ≥ 2.9) to declare a significant association between marker and trait was set based on a permutation test (*n *= 1000; *P *= 0.01) (Churchill and Doerge 1994). For the additive effect, a positive value means that the desirable allele is from the recipient parent (WTR-1), while a negative value means that the desirable allele is from the donor parent (Y-134). The graphical representation of the polymorphic SNPs and the location of the peak marker was visualized using the physical positions of each marker in PhenoGram software (Wolfe et al. 2013).

### Identification of putative candidate genes associated with ESG and ESV traits

In order to predict and identify the presence of putative candidate genes within the identified QTLs associated with ESV and ESG traits, in silico analysis was performed using the rice genome browser databases such as MSU Rice Annotation Project (RAP) (https://rapdb.dna.affrc.go.jp/) and Oryzabase database (https://shigen.nig.ac.jp/rice/oryzabase/). To view the list of identified QTLs and find the co-localized QTLs with the present QTLs, QTL Annotation Rice Online database tools (Q-TARO) (http://qtaro.abr.affrc.go.jp/) and Gramene database (https://archive.gramene.org/) were used.

## Results

### Phenotypic variation of ESG-related traits

ESG traits are the key components to improve WCA in rice. A total of ten ESG traits (1st GC, 2nd GC, GP-1, SL, RL-1, TFGS, TDGS, AFW, ADW, and VI-1) were investigated in 167 SILs and the results showed high phenotypic variation among the SILs. The mean GP-1 of WTR-1 and Y-134 was 68% and 94%, respectively, while the overall average for all SILs was 63.90% (Supplementary Table 1). More than 90% germination was observed in 11 SILs, including the donor parent Y-134. In the same set of SILs, we found the highest values for VI-1 in the range of 27.60 to 37.35. Among the ten ESG traits, the highest coefficients of variation (*CV*) values were identified in AFW (48.25%) and 1st GC after 48 h (44.05%), whereas the lowest *CV* was observed in TDGS (17.83%) and SL (18.58%). Analysis of variance (ANOVA) of these traits revealed a significant difference (*P *< 0.001) in ten traits, except RL-1 (*P* = 0.0834) and SL (*P* = 0.0137) (Table 3).

**Table 2:** Summary of markers used in genotyping 167 BC_1_F_5_ early backcross selective introgression lines (SILs) developed from an early backcross population involving Weed Tolerant Rice 1 as the recipient parent and Y-134 as the donor parent

Chromosome	Number of markers	Average distance (Kb)	Genome size covered by SNPs (Kb)	Total rice genome size (Gramene)	Coverage percentage (%)
1	77	549.3	42,297.1	43,270.9	97.7
2	53	651.2	34,511.0	35,937.3	96.0
3	61	573.3	34,970.6	36,413.8	96.0
4	35	939.8	32,891.7	35,502.7	92.6
5	55	513.4	28,236.0	29,958.4	94.3
6	74	392.5	29,043.4	31,248.8	92.9
7	76	378.4	28,758.3	29,697.6	96.8
8	56	492.1	27,559.2	28,443.0	96.9
9	47	466.3	21,918.2	23,012.7	95.2
10	35	505.7	17,699.3	23,207.3	76.3
11	58	488.1	28,309.3	29,021.1	97.5
12	50	506.7	25,333.7	27,531.9	92.0
	677	538.1	373,245.5	1124.5	93.68

^Kb-kilobase^

**Table 3:** Analysis of variance for the testing of significance of genotype effect per trait for early seed germination (ESG) traits

Trait	Abbreviation	Sum of Squares	Mean Square	F value	Pr (> F)
1st germination count	1st GC	8383.0	50.5	2.88	0.0000***
2nd germination count	2nd GC	7918.8	47.7	3.88	0.0000***
Germination percentage (%)	GP-1	128,918.7	776.6	3.79	0.0000***
Shoot length (cm)	SL	33,068.2	199.2	1.41	0.0137*
Root length (cm)	RL-1	54,759.7	329.9	1.24	0.0834
Total fresh weight of germinated seeds (g)	TFGS	8.2	0.0493	2.96	0.0000***
Total dry weight of germinated seeds (g)	TDGS	1.8	0.0107	4.06	0.0000***
Average fresh weight	AFW	0.1	0.0007	1.44	0.0099**
Average dry weight	ADW	0.01	0.0001	1.83	0.0001***
Vigor index	VI-1	23,039.1	138. 8	3.59	0.0000***

Significance codes: * 0.05 ≥ *P* ≥ 0.01; ** 0.01 ≥ *P* ≥ 0.001; ***P ≤ 0.001

In the pair-wise correlation coefficient of ESG traits, 1st GC positively and significantly correlated with 2nd GC, GP-1, TDGS, TFGS, and VI-1 (r = 0.58, r = 0.57, r = 0.49, r = 0.30, r = 0.58, *P *< 0.001), whereas a significant negative correlation was observed with AFW (r = −0.40, *P *< 0.001), ADW (r = − 0.21, *P *< 0.001), and SL (r = − 0.13, *P *< 0.01) (Supplementary Fig. 1). VI-1 was strongly associated with 1st GC, 2nd GC, GP-1, TFGS, and TDGS. The highest correlation value was recorded between the traits 2nd GC and VI-1 (r = 0.94, *P *< 0.001), GP-1 and VI-1 (r = 0.94, *P *< 0.001), and TDGS and VI-1 (r = 0.86, *P *< 0.001), whereas a negative significant correlation was observed between 1st GC with AFW (r = − 0.40) and ADW (r = − 0.21), 2nd GC with AFW (r = −0.66) and ADW (r = − 0.45), GP-1 with AFW (r = − 0.63) and ADW (r = −0.41), TFGS with AFW (r = −0.40), AFW and VI-1 (r = − 0.51), and ADW with VI-1 (r = − 0.26) at *P *< 0.001.

### Phenotypic variation of ESV-related traits

High phenotypic variation was observed in 17 ESV-related traits. The averages and *CV* values of the phenotypic traits are presented in Supplementary Table 1. Nine out of 167 SILs showed a VI-2 value of more than 130 and also had maximum GP-2, NL, PH, LCC, RFW, LFW, LDW, and RDW. Five ESV traits (GC, GP-2, LDW, RDW, and TDW) had *CV* values of 45.42%, 45.42%, 45.26%, 40.52%, and 50.08%, respectively; whereas the lowest *CV* values (< 30%) were observed in PH at 28 DAS, NT, LCC, and NL at 28 DAS. The higher *CV* suggests that the selected SILs exhibited higher genetic variability.

The summary of ANOVA (Table 4) showed significant genotypic effects on all the traits (*P* ≤ 0.001) at 1% level, except PH at 7 DAS (*P* = 0.174). Highly significant variation was observed for PH at 28 DAS, NL at 7, 14, 21, and 28 DAS, NT, RL-2, RFW, RDW, and TDW (*P *< 0.001). PH at 21 DAS showed significance only at 5% level, which indicates that very few QTLs can be located for this trait. The correlation analysis showed that all the ESV traits were significantly and positively correlated with one another except for RDW with GC and GP-2 (*P *> 0.05; r = 0.07) (Supplementary Fig. 2). Moreover, all the ESV traits were negatively correlated with weed parameters WD, WFW and WDW except for RL-2 with WD and WDW (*P *> 0.05; r = 0.11), RFW with WD (*P *> 0.05; r = 0.11), and RDW with WDW (*P *> 0.05; r = 0.1). Among the ESV traits, only eight traits had a high positive correlation observed between the traits such as TFW and LFW (r = 0.99), TDW and LDW (r = 0.98), TN and NL at 28 DAS (r = 0.88), PH at 21 DAS and NL at 14 DAS (r = 0.87), PH at 14 DAS and NL at 14 DAS (r = 0.86), TDW and TN (r = 0.80), VI-2 with GC and GP-2 (r = 0.88), PH at 7 DAS with PH at 14 DAS (r = 80), PH at 21 DAS (r = 84), NL at 7 DAS (r = 80), and NL at 14 DAS (r = 0.80) (Supplementary Fig. 2).

**Table 4:** Analysis of variance for the testing of significance of genotype effect per trait for early seedling vigor (ESV) traits

Trait	Abbreviation	Sum of squares	Mean square	F value	Pr (> F)
Germination count	GC	546.9	3.3	1.44	0.0093**
Germination percentage (%)	GP-2	21,8771.3	1317.9	1.44	0.0093**
Plant height at 7 DAS (cm)	PH at 7 DAS	6096.1	36.7	1.16	0.1742
Plant height at 14 DAS (cm)	PH at 14 DAS	17,092.2	102.1	1.49	0.0050**
Plant height at 21 DAS (cm)	PH at 21 DAS	39,066.5	235.3	1.31	0.0409*
Plant height at 28 DAS (cm)	PH at 28 DAS	38,934.8	234.6	2.19	0.0000***
Number of leaves at 7 DAS	NL at 7 DAS	130.8	0.8	1.64	0.0008***
Number of leaves at 14 DAS	NL at 14 DAS	379.6	2.3	1.62	0.0010***
Number of leaves at 21 DAS	NL at 21 DAS	2206.2	13.3	1.71	0.0003***
Number of leaves at 28 DAS	NL at 28 DAS	4559.5	27.5	2.10	0.0000***
Number of tillers	NT	259.3	1.6	2.64	0.0000***
Leaf chlorophyll content	LCC	17,313.0	104.3	1.48	0.0061**
Root length (cm)	RL-2	5372.4	32.4	1.67	0.0005***
Leaf fresh weight (g)	LFW	1801.3	10.9	1.49	0.0055**
Leaf dry weight (g)	LDW	21.3	0.1	1.56	0.0023**
Root fresh weight (g)	RFW	63.4	0.4	2.24	0.0000***
Root dry weight (g)	RDW	1.3	0.0	1.97	0.0000***
Total fresh weight (g)	TFW	2213.2	13.3	1.61	0.0012**
Total dry weight (g)	TDW	30.1	0.2	1.66	0.0006***
Vigor index	VI-2	375,273.9	2260.7	1.56	0.0023**

*DAS* days after sowing

Significance codes:*0.05 ≥ *P* ≥ 0.01; **0.01 ≥ *P* ≥ 0.001; ***P ≤ 0.001

### SNP markers for QTL mapping

**Fig. 1:**
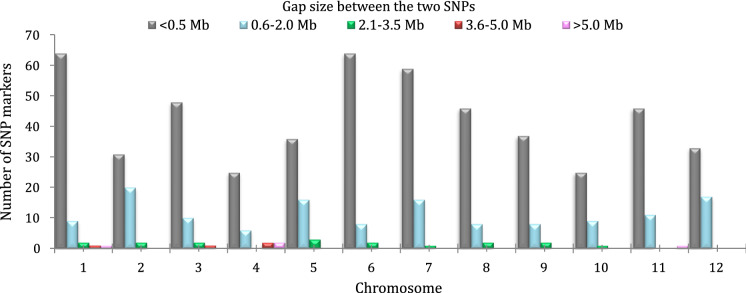
Distribution of the polymorphic single nucleotide polymorphisms (SNPs) and gaps between the two adjacent markers in all chromosomes

A total of 677 polymorphic SNP markers were detected between the parents. These markers were unevenly distributed across the 12 chromosomes, ranging from 35 SNPs on chromosome 4 and 10 to 77 SNPs on chromosome 1, with an average space of 538.1 kb between the two adjacent markers (Table 2). More than 70% of SNPs were located within 0.5 Mb of their closest neighbor (Fig. 1). In the distribution of SNPs that were generated through the 6 K SNP array, a total of eight gaps (four sites ranged from 3.6 to 5.0 Mb and another four sites ranged in more than 5.0 Mb) were found across the genome. The results of these gaps indicate the monomorphic patterns of the SNP markers shared between the two parents. The largest gaps were found on chromosome 4 (6.62 Mb), chromosome 11 (5.83 Mb), and chromosome 1 (5.39 Mb), respectively. The filtered 677 polymorphic SNPs were used to analyze the association between the ESG and ESV traits and markers.

### QTLs associated with ESG traits

**Fig. 2:**
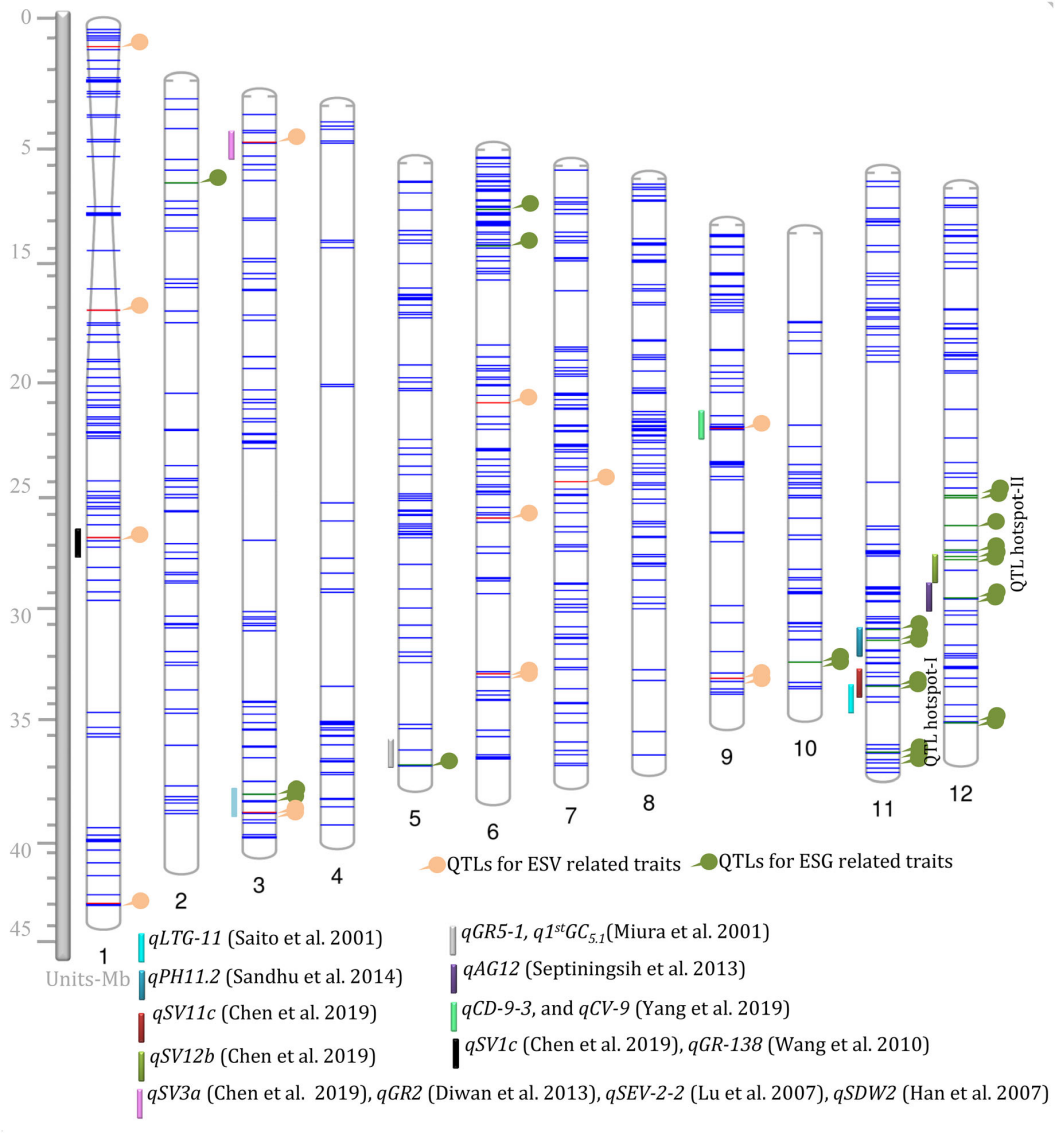
Phenogram plot showing the forty-three quantitative trait loci (QTLs) for early seed germination (ESG) and early seedling vigor (ESV) related traits in rice. The twelve chromosomes were displayed with blue color lines indicating the distribution of polymorphic single nucleotide polymorphisms (SNPs) according to the physical map. On the left side of the chromosomes were small segments of colored vertical bars representing the previously identified germination and seedling vigor QTLs that co-localized with the QTLs identified in this study. ESG QTLs previously identified were *qGR5*-*1* (Miura et al. 2001), *qPH11.2* (Sandhu et al. 2014), *qSV11c* (Chen et al. 2019), *qLTG*-*11* (Saito et al. 2001), *qSV12b* (Chen et al. 2019), and *qAG12* (Septiningsih et al. 2013). ESV QTLs previously identified were *qSV1c* (Chen et al. 2019), *qGR*-*138* (Wang et al. 2010), *qSV3a* (Chen et al. 2019), *qGR2* (Diwan et al. 2013), *qSEV*-*2*-*2* (Lu et al. 2007), *qSDW2* (Han et al. 2007), and *qCD*-*9*-*3* and *qCV*-*9* (Yang et al. 2019)

**Fig. 3:**
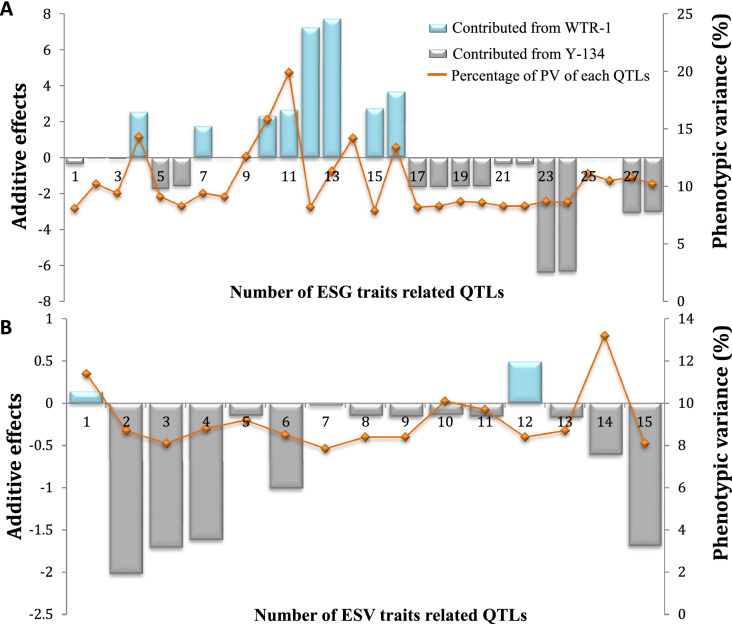
QTLs strong effects of early seed germination (ESG) and early seedling vigor (ESV) traits (LOD > 3.0) identified in the early backcross generation of selective introgression lines (SILs). The plots A and B represent the additive effect and allele effect of the 29 QTLs in ESG and 15 QTLs in ESV QTLs

A total of 28 QTLs associated with ESG traits were mapped on seven chromosomes (Fig. 2 and Table 5). Of these, 12 QTLs were located on chromosome 12, eight on chromosome 11, two each on chromosomes 3, 6, and 10, and one each on chromosomes 2 and 5, which explained their phenotypic variance (PV) ranging from 7.9% to 19.9%, respectively. Eleven QTLs had a desirable allele of WTR-1, which explained the PV of five ESG traits (1st GC, 2nd GC, GP-1, TDGS, and VI-1) ranging from 7.9% (LOD score = 2.91) to 19.9% (LOD score = 8.03). Moreover, 18 QTLs had a desirable allele contributed by Y-134 and were significantly associated with six ESG traits (1st GC, 2nd GC, GP-1, TDGS, TFGS, and VI-1). Three QTLs for 1st GC and TDGS on chromosome 11 (*q1*st *GC*_*11.1*_, *q1*st *GC*_*11.2*_, and *qTDGS*_*11.1*_) were explained by PV values of 15.8%, 19.9%, and 14.2%, respectively. The high ranges of PV of the QTLs were controlled by a desirable allele from WTR-1 (Fig. 3). Among these QTLs, one major QTL (*q1*st *GC*_*11.2*_) which exhibited the highest PV (19.9%, LOD score = 8.3) was marked by SNP_11_27994133 on chromosome 11.

**Table 5:** QTLs identified for early seed germination (ESG) traits related to weed competitive ability

QTL	Trait	Chr	Position (bp)	Peak marker	LOD	Phenotypic variance (%)	Additive effect	Parent*
*q1*st *GC*_*2.1*_	1st GC	2	4930742	SNP_2_4930742	3.0	8.1	− 0.34	Y-134
*qTDGS*_*3.1*_	TDGS	3	33713486	SNP_3_33713486	3.9	10.2	0.03	WTR-1
*qTFGS*_*3.1*_	TFGS	3	33713486	SNP_3_33713486	3.5	9.4	− 0.06	Y-134
*q1*st *GC*_*5.1*_	1st GC	5	29061672	SNP_5_29061672	5.6	14.3	2.52	WTR-1
*q1*st *GC*_*6.1*_	1st GC	6	2871279	SNP_6_2871279	3.4	9.1	− 1.73	Y-134
*q1*st *GC*_*6.2*_	1st GC	6	4641044	SNP_6_4641044	3.1	8.3	− 1.60	Y-134
*q1*st *GC*_*10.1*_	1st GC	10	20723502	SNP_10_20723502	3.5	9.4	1.74	WTR-1
*qTDGS*_*10.1*_	TDGS	10	20723502	SNP_10_20723502	3.4	9.1	0.03	WTR-1
*q1*st *GC*_*11.1*_	1st GC	11	22546707	SNP_11_22546707	6.2	15.8	2.30	WTR-1
*q1*st *GC*_*11.2*_	1st GC	11	27994133	SNP_11_27994133	8.0	19.9	2.65	WTR-1
*q2*nd *GC*_*11.1*_	2nd GC	11	24779246	SNP_11_24779246	4.3	11.3	1.93	WTR-1
*qGP*-*1*_*11.1*_	GP-1	11	22044151	SNP_11_22044151	3.0	8.2	7.24	WTR-1
*qGP*-*1*_*11.2*_	GP-1	11	24779246	SNP_11_24779246	4.3	11.3	7.73	WTR-1
*qTDGS*_*11.1*_	TDGS	11	27994133	SNP_11_27994133	5.5	14.2	0.03	WTR-1
*qVI*-*1*_*11.1*_	VI-1	11	22546707	SNP_11_22546707	2.9	7.9	2.73	WTR-1
*qVI*-*1*_*11.2*_	VI-1	11	27994133	SNP_11_27994133	5.2	13.4	3.66	WTR-1
*q1*st *GC*_*12.1*_	1st GC	12	14835375	SNP_12_14835375	3.0	8.2	− 1.63	Y-134
*q1*st *GC*_*12.2*_	1st GC	12	17788006	SNP_12_17788006	3.1	8.3	− 1.62	Y-134
*q1*st *GC*_*12.1*_	1st GC	12	14936674	SNP_12_14936674	3.0	8.3	− 0.37	Y-134
*q1*st *GC*_*12.2*_	1st GC	12	17443323	SNP_12_17443323	3.0	8.3	− 0.37	Y-134
*q2*nd *GC*_*12.1*_	2nd GC	12	16286946	SNP_12_16286946	3.2	8.7	− 1.59	Y-134
*q2*nd *GC*_*12.2*_	2nd GC	12	17902839	SNP_12_17902839	3.2	8.6	− 1.58	Y-134
*qGP*-*1*_*12.1*_	GP-1	12	16286946	SNP_12_16286946	3.2	8.7	− 6.37	Y-134
*qGP*-*1*_*12.2*_	GP-1	12	17902839	SNP_12_17902839	3.2	8.6	− 6.32	Y-134
*qTFGS*_*12.1*_	TFGS	12	19786034	SNP_12_19786034	4.2	11.1	− 0.03	Y-134
*qTFGS*_*12.2*_	TFGS	12	25792416	SNP_12_25792416	4.0	10.5	− 0.03	Y-134
*qVI*-*1*_*12.1*_	VI-1	12	16287347	SNP_12_16287347	4.1	10.8	− 3.05	Y-134
*qVI*-*1*_*12.2*_	VI-1	12	25792416	SNP_12_25792416	3.9	10.2	− 3.00	Y-134

*Parent- contributing desirable allele. For the additive effect, a positive value means that the desirable allele is from the recipient parent (WTR-1), while a negative value means that the desirable allele is from the donor parent (Y-134). Abbreviations: Chr, chromosome; LOD, logarithm of the odds; 1st GC, 1st germination count; 2nd GC, 2nd germination count; GP-1, germination percentage; TFGS, total fresh weight of germinated seeds; TDGS, total dry weight of germinated seeds; and VI-1, vigor index

For TFGS, two major QTLs (*qTFGS*_*12.1*_ and *qTFGS*_*12.2*_) on chromosome 12 and one minor QTL (*qTFGS*_*3.1*_) on chromosome 3 were detected with a desirable allele contributed by the donor parent Y-134. Together, the TFGS QTLs explained 11.2% of PV. Three major QTLs on chromosomes 11 and 12 that controlled the ESG-related trait VI-1 were *qVI*-*1*_*11.2*_, *qVI*-*1*_*12.1*_, and *qVI*-*1*_*12.2*_, with PV of 13.4%, 10.8%, and 10.2% and a LOD score of 5.21, 4.16, and 3.9, respectively (Table 5 and Fig. 3). The fourth QTL (*qVI*-*1*_*11.1*_) explained the PV of 7.9% and had a LOD score of 2.98. The additive effect of the QTLs on chromosome 11 (*qVI*-*1*_*11.1*_ and *qVI*-*1*_*11.2*_) was positive, implying that the desirable alleles were contributed by WTR-1, whereas the additive effect of the QTLs on chromosome 12 (*qVI*-*1*_*12.1*_ and *qIV*-*1*_*12.2*_) was negative, and was contributed by Y-134.

### QTLs associated with ESV traits

In the present study, 15 QTLs associated with ESV-related traits (PH at 7, 14, 21, and 28 DAS, NL at 7 and 21 DAS, RFW, and RDW) were mapped on five different chromosomes (1, 3, 6, 7, and 9). Five QTLs associated with PH at 7, 14, 21, and 28 DAS were located on chromosomes 1, 3, and 9 (Table 6 and Fig. 2) and explained 42.2% of PV. On chromosomes 1 and 9, two QTLs for PH at 14 DAS (*qPH*-*14*_*1.1*_, PV = 8.7%, and *qPH*-*14*_*9.1*_, PV = 8.1%) were marked by SNP_1_42397588 and SNP_9_9834510. The other three remaining QTLs (*qPH*-*7*_*3.1*_, *qPH*-*21*_*1.1*_, and *qPH*-*28*_*1.2*_) were linked with PH at 7, 21, and 28 DAS, with LOD scores of 3.18 (PV = 8.5%), 3.06 (PV = 8.1%), and 3.30 (PV = 8.8%), respectively. The additive effect indicated that Y-134 contributed to the desirable alleles (Fig. 3).

**Table 6:** QTLs identified for early seedling vigor (ESV) traits related to weed competitive ability

No.	QTL	Trait	Chr	Position (bp)	Peak marker	LOD	Phenotypic variance (%)	Additive effect	Parent*
1	*qNL*-*7*_*1.1*_	NL at 7 DAS	1	1022215	SNP_1_1022215	4.3	11.4	0.14	WTR-1
2	*qPH*-*14*_*1.1*_	PH at 14 DAS	1	42397588	SNP_1_42397588	3.2	8.7	− 2.02	Y-134
3	*qPH*-*21*_*1.1*_	PH at 21 DAS	1	13777831	SNP_1_13777831	3.0	8.1	− 1.71	Y-134
4	*qPH*-*28*_*1.2*_	PH at 28 DAS	1	24722142	SNP_1_24722142	3.3	8.8	− 1.62	Y-134
5	*qNL*-*7*_*3.1*_	NL at 7 DAS	3	34568654	SNP_3_34568654	3.4	9.2	− 0.15	Y-134
6	*qPH*-*7*_*3.1*_	PH at 7 DAS	3	2230854	SNP_3_2230854	3.1	8.5	− 1.01	Y-134
7	*qRDW*_*3.1*_	RDW	3	34568654	SNP_3_34568654	2.9	7.85	− 0.03	Y-134
8	*qNL*-*7*_*6.1*_	NL at 7 DAS	6	12183428	SNP_6_12183428	3.1	8.4	− 0.15	Y-134
9	*qNL*-*7*_*6.2*_	NL at 7 DAS	6	17750942	SNP_6_17750942	3.1	8.4	− 0.16	Y-134
10	*qNL*-*7*_*6.3*_	NL at 7 DAS	6	25277863	SNP_6_25277863	3.7	10.1	− 0.14	Y-134
11	*qRFW*_*6.1*_	RFW	6	25277863	SNP_6_25277863	3.6	9.7	− 0.16	Y-134
12	*qNL*-*21*_*7.1*_	NL at 21 DAS	7	15241178	SNP_7_15241178	3.1	8.4	0.49	WTR-1
14	*qNL*-*21*_*9.1*_	NL at 21 DAS	9	21885499	SNP_9_21885499	3.2	8.7	− 0.61	Y-134
13	*qNL*-*7*_*9.1*_	NL at 7 DAS	9	21896910	SNP_9_21896910	5.0	13.2	− 0.17	Y-134
15	*qPH*-*14*_*9.1*_	PH at 14 DAS	9	9834510	SNP_9_9834510	3.0	8.1	− 1.69	Y-134

*Parent- contributing desirable allele. For the additive effect, a positive value means that the desirable allele is from the recipient parent (WTR-1), while a negative value means that the desirable allele is from the donor parent (Y-134). Abbreviations: Chr, chromosome; LOD, logarithm of the odds; DAS, days after sowing; NL at 7 DAS, number of leaves at 7 DAS; NL at 21 DAS, number of leaves at 21 DAS; PH at 7 DAS, plant height at 7 DAS; PH at 14 DAS, plant height at 14 DAS; PH at 21 DAS, plant height at 21 DAS; PH at 28 DAS, plant height at 28 DAS; RFW, root fresh weight; and RDW, root dry weight

To date, there have been no reports on QTLs associated with NL at different growth stages. In this study, eight novel QTLs associated with NL were identified on chromosomes 1, 3, 6, 7, and 9 (Table 6). Among the eight novel QTLs, one major QTL (*qNL*-*7*_*1.1*_) and another minor QTL (*qNL*-*21*_*7.1*_) were contributed by the WTR-1 allele and the remaining six QTLs were influenced by a desirable allele coming from the donor parent Y-134 (Table 6 and Fig. 3). Among the novel QTLs, three were major QTLs and were associated with NL on chromosome 1 (*qNL*-*7*_*1.1*_, PV = 11.4%), chromosome 6 (*qNL*-*7*_*6.3*_, PV = 10.1%), and chromosome 9 (*qNL*-*7*_*9.1*_, PV = 13.2%), with LOD scores of 4.30, 3.78, and 5.04, respectively. The major QTL (*qNL*-*7*_*1.1*_*)* on chromosome 1 was marked by SNP_1_1022215 and exhibited the highest PV (11.4%), with a LOD score of 4.3 (Table 6).

### Hotspots and co-localized QTLs for ESG and ESV traits

QTLs associated with WCA-related traits were identified in two hotspot regions on chromosomes 11 and 12. Eight QTLs were located on chromosome 11 at the position from 22.4 Mb to 27.9 Mb, and it was labeled as “QTL hotspot I”, which covers a total genomic length of 5.5 Mb. Similarly, chromosome 12 contained a total of 12 QTLs that were located at the position from 14.8 Mb to 25.7 Mb, and this was labeled as “QTL hotspot II”, with a total coverage length of 10.9 Mb (Fig. 2). QTL hotspot I was associated with five ESG-related traits (GP-1, 1st GC, VI-1, 2nd GC, and TDGS) grouped together with an average PV of 12.65%, whereas QTL hotspot II contained five ESG-related traits (1st GC, 2nd GC, GP-1, VI-1, and TFGS) effectively showing the average PV of 9.19%. Interestingly, chromosome 11 had an additive effect contributed by a WTR-1 allele. In contrast to chromosome 11, the additive allele from Y-134 contributed to all the QTLs on chromosome 12.

Taken together, 58.6% and 26.6% of the ESG and ESV QTLs were co-localized. ESV traits related to two QTLs (*qNL*−*7*_*6.3*_ and *qRFW*-*7*_*6.1*_) on chromosome 6 were marked with SNP_6_25277863 at the position of 25.2 Mb and two QTLs (*qRDW*_*3.1*_ and *qNL*-*7*_*3.1*_) on chromosome 3 were marked by SNP_3_34568654 at the position of 34.5 Mb and were co-localized. Both of these QTLs were contributed by a Y-134 allele. For ESG traits, three QTLs (*q1*st *GC*_*11.2*_, *qTDGS*_*11.1*_, and *qVI*-*1*_*11.2*_) at 27.9 Mb, two QTLs (*q2*nd *GC*_*11.1*_ and *qGP*-*1*_*11.2*_) at 24.7 Mb, and two QTLs (*q1*st *GC*_*11.1*_ and *qVI*-*1*_*11.1*_) at 22.5 Mb were co-localized on chromosome 11; whereas two QTLs (*q1st GC*_*10.1*_ and *qTDGS*_*10.1*_) at the position of 20.7 Mb were co-localized on chromosome 10. These co-localized QTLs were contributed by a WTR-1 allele. Similarly, on chromosome 12, two QTLs (*q2*nd *GC*_*12.1*_ and *qGP*-*1*_*12.1*_) at 16.2 Mb, two QTLs (*q2*nd *GC*_*12.2*_ and *qGP*-*1*_*12.2*_) at 17.9 Mb, and two other QTLs (*qTFGS*_*12.2*_ and *qVI*-*1*_*12.2*_) at 25.7 Mb were co-localized. The hotspot co-localized QTLs on chromosome 12 was associated with a desirable allele from Y-134. Interestingly, studies on the hotspot QTL regions revealed that the alleles were associated with both parents, and this indicated that a wide range of the molecular and phenotypic diversity of ESG and ESV traits related to WCA existed among the SILs.

### Putative candidate genes associated with ESG and ESV traits

The QTL hotspot regions and co-localized QTLs on chromosomes 11 and 12 were used to analyze the candidate genes for WCA in rice. A total of five possible genes on chromosome 11 and eight genes on chromosome 12 were identified (Table 7). Out of 13 putative genes, two were hypothetical, six were putative proteins, and the remaining five genes were well reported to be involved in multiple functions related to biotic and abiotic stress tolerance in rice. On chromosome 11, GP is associated with pentatricopeptide repeat (PPR) domain and three other co-localized QTLs (*q1*st *GC*_*11.2*_, *qTDGS*_*11.1*_, and *qVI*-*1*_*11.2*_) were associated with tetra-tricopeptide repeat (TPR) domain at 22 Mb and 27.9 Mb positions, respectively. Earlier reports of Gothandam et al. (2005), Lin et al. (2015) and Yu et al. (2016) have suggested that PPR domain are located on different chromosomes. These domains are functionally related to chloroplast development, photosynthesis, seedling lethality during the early leaf growth stage, embryogenesis, seed development, and cytoplasmic male sterility (Sosso et al. 2012; Gong et al. 2014; Huang et al. 2015; Lin et al. 2015; Sharma and Pandey 2016). In addition to that, TPR-containing protein has been involved in several functions such as cell cycle, regulation of gibberellins (GAs), seed development, hybrid sterility, endosperm development, and seed setting (Awasthi et al. 2012; Lin et al. 2015). Similarly, other co-localized QTLs (*q1*st *GC*_*11.1*_ and *qVI*-*1*_*11.1*_) at 22.5 Mb on chromosome 11 are found within the range of 4kbs of *Os11g38010*. This locus has been reported to encode TPX2 homolog, which is considered to be involved in the organization of microtubule spindle formation during cell division (Guo et al. 2008). Two hypothetical and putative expressed loci (*Os11g41240* and *Os11g41320*) were found to be associated with the two QTLs (*q2*nd *GC*_*11.1*_ and *qGP*-*1*_*11.2*_) mapped in this study.

**Table 7:** Possible putative candidate genes identified from the hotspot QTL regions for weed competitive ability (WCA) traits in rice

No.	Chromosome	CDS coordinates (5′–3′)		Name of the loci/gene	Locus	Nucleotide length (bp)
		Start	End			
1	11	22040368	22044971	Pentatricopeptide repeat domain containing protein, putative, expressed	LOC_Os11g37330.1	2085
2	11	22550213	22554890	Targeting protein for Xklp2, putative, expressed	LOC_Os11g38010.1	1224
3	11	24727308	24727862	ATBPM6, putative	LOC_Os11g41240.1	555
4	11	24780333	24781296	Hypothetical protein	LOC_Os11g41320.1	240
5	11	27997070	27991732	Tetratricopeptide repeat domain containing protein, expressed	LOC_Os11g46230.1	2094
6	12	14845431	14838519	SAP domain containing protein, expressed	LOC_Os12g25640.1	537
7	12	14933967	14933192	Expressed protein	LOC_Os12g25760.1	354
8	12	16278035	16279881	Hypothetical protein	LOC_Os12g27650.1	495
9	12	17441549	17443641	ZmEBE-1 protein, putative, expressed	LOC_Os12g29370.1	909
10	12	17786025	17788513	The protein of unknown function DUF502 domain-containing protein, expressed	LOC_Os12g29750.1	825
11	12	17900930	17903046	Nodulin, putative, expressed	LOC_Os12g29950.1	1800
12	12	19779505	19786587	Flavin monooxygenase, putative, expressed	LOC_Os12g32750.1	1347
13	12	25790709	25796385	Expressed protein	LOC_Os12g41670.1	17,331

On chromosome 12, QTL *q1*st *GC*_*12.1*_ is associated with SAP domain-containing protein, which is functionally related to stress-associated proteins and are involved in regulating GA and ABA signaling in response to abiotic stresses (Huang et al. 2008; Giri et al. 2011; Zhang et al. 2015; Kothari et al. 2016). Growth hormone regulations are vital to seedling growth and development. The QTL *qTFGS*_*12*_ is associated with *Os12g32750* at the position of 19.7 Mb. These loci are responsible for flavin monooxygenases, which have a significant functional role in the tryptophan (Trp)-dependent indole-acetic acid synthesis for auxin biosynthetic pathways for the improvement of quick response to early seedling growth and root tip development (Yamamoto et al. 2007; Fujino et al. 2008; Yi et al. 2013). Interestingly, we identified six loci (*Os12g27650, Os12g29950 Os12g41670, Os12g25760, Os12g29370,* and *Os12g29750)* that were associated with unknown and hypothetical proteins in the rice genome databases (Table 7).

## Discussion

Breeding for WCA in rice is essential for the development of DSR varieties under both dry and wet-seeded methods. WCA provides rapid early growth toward crop establishment and suppression of weed growth in DSR and aerobic rice ecosystem. A significant positive correlation in traits such as germination percentage, germination count, and vigor index indicates a strong positive relationship with field emergence and seedling establishment, which are the favorable traits for WCA in rice. The Green Super Rice (GSR) breeding strategy (Zhang 2007; Ali et al. 2017, 2018; Dimaano et al. 2017) helped in developing the mapping population ideally selected for WCA traits in a systematic manner with progeny testing. Germination percentage of WTR-1 and Y-134 was 94% and 68%, respectively; whereas the germination percentage of the SILs showed a significant variation from 2% to 98%. The extreme phenotypic variation in germination indicates that transgressive segregation took place in the population. This also suggests that both parents possess positive QTLs and genes for WCA and that WCA is controlled by multiple QTLs and genes in rice. Based on the vigor index (> 120) and germination percentage (> 90) values, 17 SILs and 10 SILs were identified as promising SILs possessing ESV and ESG traits, respectively. Five SILs with ESG and 15 SILs with ESV were shown to maintain a higher germination percentage and vigor index than the parents. Two SILs were commonly identified as promising SILs in both experiments of ESG and ESV. These promising SILs can be useful in breeding programs for the development of rice cultivars with WCA.

Mahender et al. (2015) reviewed and mentioned 38 QTLs that were associated with germination rate, germination index, and germination percentage and germination time in different genetic backgrounds of mapping populations. In the present study, the major QTL *qGP*-*1*_*11.2*_ coincides with germination percentage, germination rate, and germination index on chromosome 11 and was also observed in the genetic background of recombinant inbred line (RIL) mapping populations of Daguandao (*japonica)* and IR28 *(indica),* respectively (Wang et al. 2010). Several QTLs for ESG-related traits (1st GC and 2nd GC) were previously reported: *qLTG*-*2* on chromosome 2 (Miura et al. 2001), *qGR3*-*1* and *qGR3*-*3* on chromosome 3 (Cui et al. 2002a), *qLTG*-*4*-*2*, *qLTG*-*4*-*1*, and *qGP*-*4* on chromosome 4 (Miura et al. 2001; Wang et al. 2010), *qGR5*-*1* and *qLTG*-*5* on chromosome 5 (Miura et al. 2001; Cai and Morishima 2002; Cui et al. 2002a, b), *qGR6*-*2* on chromosome 6 (Cui et al. 2002a; Wang et al. 2010), *qGR7*-*1* on chromosome 7 (Cui et al. 2002a), and *qLTG*-*11*, *qGR*-*11*, and *qGI*-*11* on chromosome 11 (Saito et al. 2001; Wang et al. 2010) in the diverse sets of rice mapping populations such as RILs, BILs, and DHs. Some QTLs for ESG-related traits identified in this study co-localized with previously reported QTLs, e.g., *q1*st *GC*_*5.1*_ co-localized with *qGR5*-*1* (Miura et al. 2001) on chromosome 5 (Fig. 2). On chromosome 11, *q1*st *GC*_*11.1*_, *qGP*-*1*_*11.2*_ and *q2*nd *GC*_*11.1*_, and *qGP*-*1*_*11.2*_ co-localized with *qPH11.2* (Sandhu et al. 2014), *qSV11c* (Chen et al. 2019), and *qLTG*-*11* (Saito et al. 2001), respectively. On chromosome 12, *q1*st *GC*_*12.2*_ and *qVI*-*1*_*12.1*_ co-localized with *qSV12b* (Chen et al. 2019); while *q2*nd *GC*_*12.2*_ and *qGP*-*1*_*12.2*_ co-localized with *qAG12* (Septiningsih et al. 2013) (Fig. 2). Therefore, the co-localization of these QTLs will provide a genetic basis underlying the correlation among the traits. Chromosomes 11 and 12 contained more than five traits related to ESG, which indicates that they are actively associated with rapid seedling growth in rice. Out of 28 identified QTLs for ESG traits, eight GP and VI QTLs were reported earlier (Diwan 2006; Hayashi et al. 2008; Yang et al. 2010; Diwan et al. 2013; Anandan et al. 2016; Singh et al. 2017), while the remaining 20 QTLs were novel.

Numerous morphological and physiological key traits are involved in ESV that determines the improvement of seedling growth and grain yield component traits (Mahender et al. 2015). Based on the comprehensive ESV QTL analysis, five chromosomes were harboring multiple trait-associated QTLs and co-localized promising QTLs for ESV in rice (Mahender et al. 2015). A total of 15 morpho-physiological traits such as germination rate, shoot and root length, shoot fresh and dry weight, seedling early vigor, leaf area, reducing sugar content and field vigor were found on chromosome 1 (Yan et al. 1998; Marri et al. 2005; Lu et al. 2007; Zhou et al. 2007; Namuco et al. 2009; Wang et al. 2010; Cheng et al. 2013; Diwan et al. 2013; Dang et al. 2014). Seven traits like shoot length, shoot dry weight, germination rate, root length, seedling early vigor, seedling fresh weight, and coleorhiza length were found on chromosome 3 (Zhi-Hong et al. 2005; Lu et al. 2007; Zhou et al. 2007; Cheng et al. 2013). Similarly, chromosome 6 was associated with seven traits such as total dry weight, germination rate, shoot dry weight, reducing sugar content, seed size, germination percentage, and shoot length (Wang et al. 2010; Xie et al. 2014). Three traits such as germination rate, germination index and shoot length were found on chromosome 7 (Mei et al. 2005; Dang et al. 2014) and four traits like root activity, shoot dry weight, seed size and leaf area were found on chromosome 9 (Cui et al. 2002a) that were responsible for more than three ESV traits in different mapping populations.

Some QTLs for ESV-related traits identified in this study co-localized with previously reported QTLs such as *qSV1c* (Chen et al. 2019) and *qGR*-*138* (Wang et al. 2010) co-localizing with *qPH*-*28*_*1.2*_ on chromosome 1; *qSV3a* (Chen et al. 2019), *qGR2* (Diwan et al. 2013), *qSEV*-*2*-*2* (Lu et al. 2007), and *qSDW2* (Han et al. 2007) co-localizing with *qPH*-*7*_*3.1*_ on chromosome 3; and *qCD*-*9*-*3* and *qCV*-*9* (Yang et al. 2019) co-localizing with *qPH*-*14*_*9.1*_ on chromosome 9 (Fig. 2). Four QTLs contributing to NL at 7 DAS and PH at 14, 21, and 28 DAS overlapped with earlier reported QTLs related to 15 ESV traits on chromosome 1 at the physical position from 29.7 cM (RM259) to 146.1 cM (RM315). These ESV traits associated with QTLs were earlier reported by using a diverse genetic resource of rice accessions and biparental mapping populations (Yan et al. 1998; Marri et al. 2005; Cairns et al. 2009; Diwan et al. 2013; Dang et al. 2014). QTLs for PH at 14, 21 and 28 DAS on chromosome 1 shared common genomic regions associated with SL, which was reported by Li et al. (2006), Namuco et al. (2009), Yan et al. (1998) and Zhou et al. (2007). Similarly, Diwan et al. (2013) identified six QTLs for SL in the 18.8 to 71.6 cM region, which showed 10% to 15% significant PV. The identical chromosomal segment of the genomic region was controlling other ESV-related traits according to Redona and Mackill (1996) and Zhang et al. (2004). Interestingly, the α-amylase gene *amy1B/A* is located at 13 cM (Temnykh et al. 2001), which is near the novel QTL *NL*-*7*_*1.1*_ on chromosome 1. This gene may influence a higher germination rate and faster seedling growth at the early stage through the degradation of starch energy sources by *α*-*amylase* in the rice embryo. The same genomic regions were controlling ESV traits and were frequently detected across other mapping populations of *O. rufipogon* and *japonica* cultivar ‘Jefferson’ (Thomson et al. 2003).

On chromosome 3, three ESV-related QTLs (*qNL*-*7*_*3.1*_, *qPH*-*7*_*3.*1_, and *qRDW*_*3.1*_) influence WCA in rice. The NL at 7 DAS and RDW QTLs overlapped in the same genomic region marked by SNP_3_34568654. In other reports, several ESV-related traits such as shoot length, shoot dry weight, germination rate, seedling early vigor, seedling fresh weight, and coleorhiza length were also mapped on chromosome 3 and were identified from two different RIL populations derived from Lemont/Teqing (Zhi-Hong et al. 2005; Lu et al. 2007; Zhou et al. 2007) and Jiucaiqing/IR26 (Cheng et al. 2013), a doubled-haploid population of CT9993/IR62266 (Kanbar et al. 2006), BC_3_F_4_ lines from Swarna/Moroberekan (Singh et al. 2017), and a natural diverse germplasm of rice accessions (Dang et al. 2014). Further, in support to the findings on chromosome 3, Singh et al. (2017) recently reported a QTL hotspot in the chromosome 3 region that had three possible candidate genes (*Os03g0236200*, *Os03g0324300*, and *Os03g0428700*). These genes are involved in different roles for the development of young seedling, mesocotyl length, coleoptile elongation, and increasing physiological activity via changes in the ethylene signaling mechanism in cell differentiation, elongation, enzyme activities, and expansion genes, which demonstrate early seedling emergence and growth development.

On chromosome 6, four QTLs (*qNL*-*7*_*6.1*_, *qNL*-*7*_*6.2*_, *qNL*-*7*_*6.3*_, and *qRFW*_*6.1*_) were mapped, while two QTLs (*qNL*-*7*_*6.3*_ and *qRFW*_*6.1*_) overlapped with the same genetic marker of SNP_6_25277863. In other reports, the QTLs located in the region of 48.7 to 101.1 cM of chromosome 6 were associated with six ESV-related traits such as germination rate, shoot dry weight, reducing sugar content, seed size, germination percentage, and shoot length in two different recombinant populations from ZS97/MH63 (Xie et al. 2014) and Daguandao/IR28 (Wang et al. 2010). The same genetic region is very close to the other QTLs: *qGW*-*6* for the 1000-seed weight (Wan et al. 2005), *sd6.1* for seed dormancy (Li et al. 2006), *qEV*_*6.1*_ for early vigor, *qEUE*_*6.1*_ for early uniform emergence, and *qSHL*_*6.1*_ for shoot length at 21 DAS (Singh et al. 2017).

On chromosome 7, the QTL controlling NL at 21 DAS (*qNL*-*21*_*7.1*_) was associated with previous reports on six ESV-related traits such as shoot length and tiller number, weight of mobilized seed reserve, leaf area, germination rate, and germination index in the QTL mapping studies from RILs (Mei et al. 2005; Zhi-Hong et al. 2005; Wang et al. 2010; Cheng et al. 2013), BC_3_F_3_ (Namuco et al. 2009), and natural germplasms (Dang et al. 2014). Three QTLs controlling NL at 7 and 21 DAS and PH at 14 DAS (*qNL*-*7*_*9.1*_, *qPH*-*14*_*9.1*_, and *qNL*-*21*_*9.1*_) were mapped on chromosome 9. The physical position of the novel QTL located on chromosome 9 at 87.5 and 39.3 cM was close to the genomic region and was associated with three ESV traits such as shoot dry weight, root activity, and seed weight in the genetic background of RILs (Zhenshan 97 and Minghui 63) reported by Cui et al. (2002a). The co-localization of all the QTLs related to ESV morphological traits such as germination-attributed traits, shoot and root length, fresh and dry weight of shoot and root, and mesocotyl length, and physiological traits such as reducing sugar, photosynthetic performance, leaf area, chlorophyll content, amylase activity, nitrate reductase, peroxidase, growth regulation hormones (abscisic acid, auxin, and gibberellins), and antioxidant enzymes (glutamic acid decarboxylase activity) located in the same genetic region provided valuable genomic information for improving WCA in rice.

To date, there is no published evidence on QTLs for rice WCA traits such as periodic germination counts, germinated seedlings with fresh and dry weight, number of leaves at 7 and 21 DAS, and average fresh weight of seedlings. Here, we identified novel QTLs for these traits. The novel and co-localized QTLs on chromosomes 3, 11, and 12 were associated with multiple traits, such as 1st GC, 2nd GC, VI-1, GP-1, TDGS, and TFGS. These QTLs were strongly correlated with ESG and ESV traits. Therefore, further high-resolution mapping studies are required for the validation of the expression and pleiotropic effect of these QTLs. However, the majority of early QTL studies have reported that multiple ESV traits are controlled by the same genomic region of reported chromosomes 1, 3, 5, 6, 9, 11, and 12 (Miura et al. 2001; Cai and Morishima 2002; Cui et al. 2002a, b; Kanbar et al. 2006; Koseki et al. 2010; Cheng et al. 2013; Diwan et al. 2013; Dang et al. 2014; Mahender et al. 2015; Singh et al. 2017). The novel QTLs accounting for a higher LOD and PV could be a potential target in future breeding programs and subsequent studies are needed to find the candidate genes and alleles for the strong association to understand the physiological and molecular mechanism conferring WCA.

## Conclusions

WCA is a vital target trait that needs to be considered by rice breeders in developing DSR varieties. A systematic GSR breeding strategy involving early backcross breeding with phenotypic selection and progeny testing for WCA traits led to the development of a population for genetic analysis. This approach led to the identification of donors for QTLs, and genes for many of the WCA traits essential to the development of rice varieties for DSR and aerobic systems. The identification of QTLs for ESG and ESV is critical for accelerating breeding programs for weed competitive rice cultivars. Therefore, the present study attempted to identify the chromosomal regions and the QTLs governing these traits. The overall WCA QTLs were contributed by both parents, WTR-1 and Y-134. Out of 43 QTLs, 30 were contributed by a desirable allele from Y-134, whereas 13 were contributed by a desirable allele from WTR-1. The frequency of ESG and ESV traits associated with QTLs showed continuous segregation, and it is controlled by multiple QTLs and genes in rice. As many as 28 novel QTLs were identified from a total of 43 QTLs that govern the genetic mechanism of WCA. Among these, the majority of the QTLs were associated with two hotspot QTL regions: on chromosome 11 with eight QTLs detected and on chromosome 12 with 12 QTLs detected, and a few of them were co-localized QTLs. The hotspot and co-localized QTL regions could have a higher potential role in the improvement of WCA. In silico analysis of the QTL hotspots on chromosomes, 11 and 12 regarding their respective genomic positions revealed that two hypothetical and six putative candidate genes were located in these hotspots. Further investigation to fine-map and use of cloning strategies are required to identify novel candidate genes for WCA in rice. The two promising SILs that were identified to have both the ESG and ESV traits could be directly used in DSR breeding programs. The prominent QTLs from the promising SILs for WCA traits can be used in the development of functional markers and QTL pyramiding with desirable genetic backgrounds. These markers could be further employed for the introgression of genes/QTLs into elite rice cultivars through a marker-assisted selection in the plant breeding program for rice varieties with WCA.

## Electronic supplementary material

Below is the link to the electronic supplementary material.

Supplementary material 1 (DOC 1138 kb)

Supplementary material 2 (DOC 68 kb)
