# Supplementary material for: Identification of quantitative trait loci governing early germination and seedling vigor traits related to weed competitive ability in rice
Source: Euphytica. 2020 Sep 19;216(10):159. doi: 10.1007/s10681-020-02694-8 (PMC7510932; doi:10.1007/s10681-020-02694-8)
Supplement: Supplementary file 1 — Supplementary material 1 (DOC 1138 kb) [file 10681_2020_2694_MOESM1_ESM.doc]

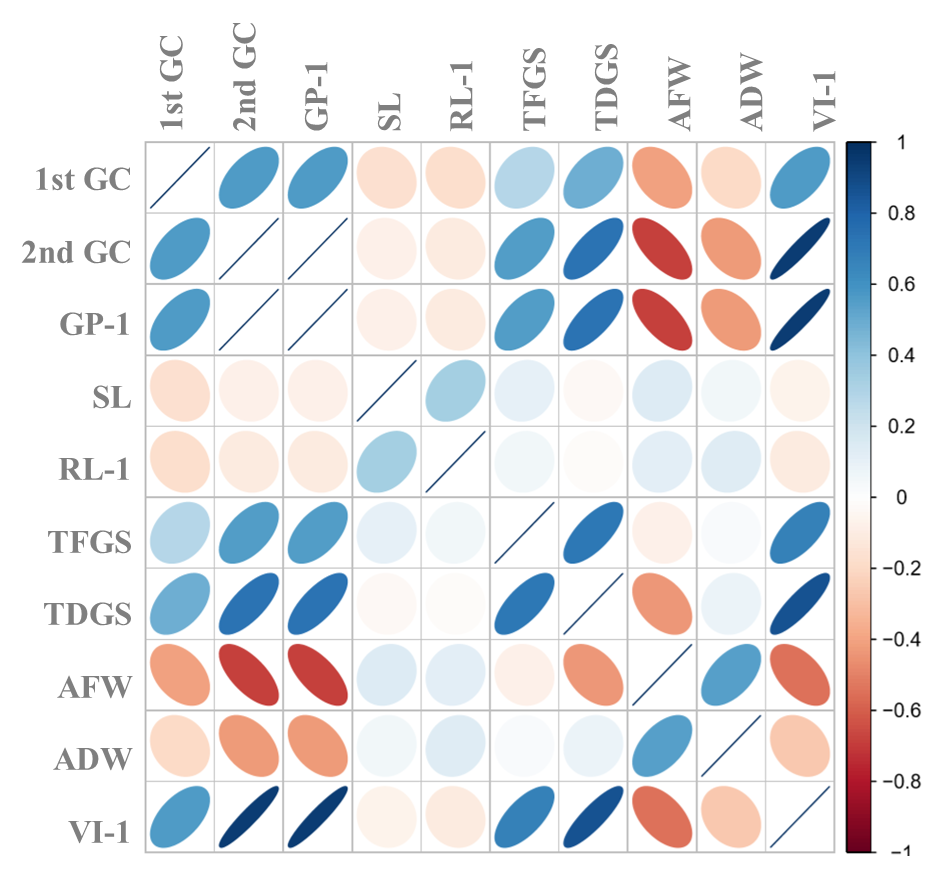


**Supplemental Fig. 1 Correlation matrix of early seed germination (ESG) related traits.** The scale represents the values of the Pearson coefficient between the ESG-related traits using the mean value of each trait. The color of bars (red, blue, and white) indicates the positive, negative, and no significant correlations between the traits, respectively. Abbreviations: 1st GC, 1st germination count; 2nd GC, 2nd germination count; GP-1, germination percentage; SL, shoot length; RL-1, root length; TFGS, total fresh weight of germinated seeds; TDGS, total dry weight of germinated seeds; AFW, average fresh weight; ADW, average dry weight; and VI-1, vigor index.

**
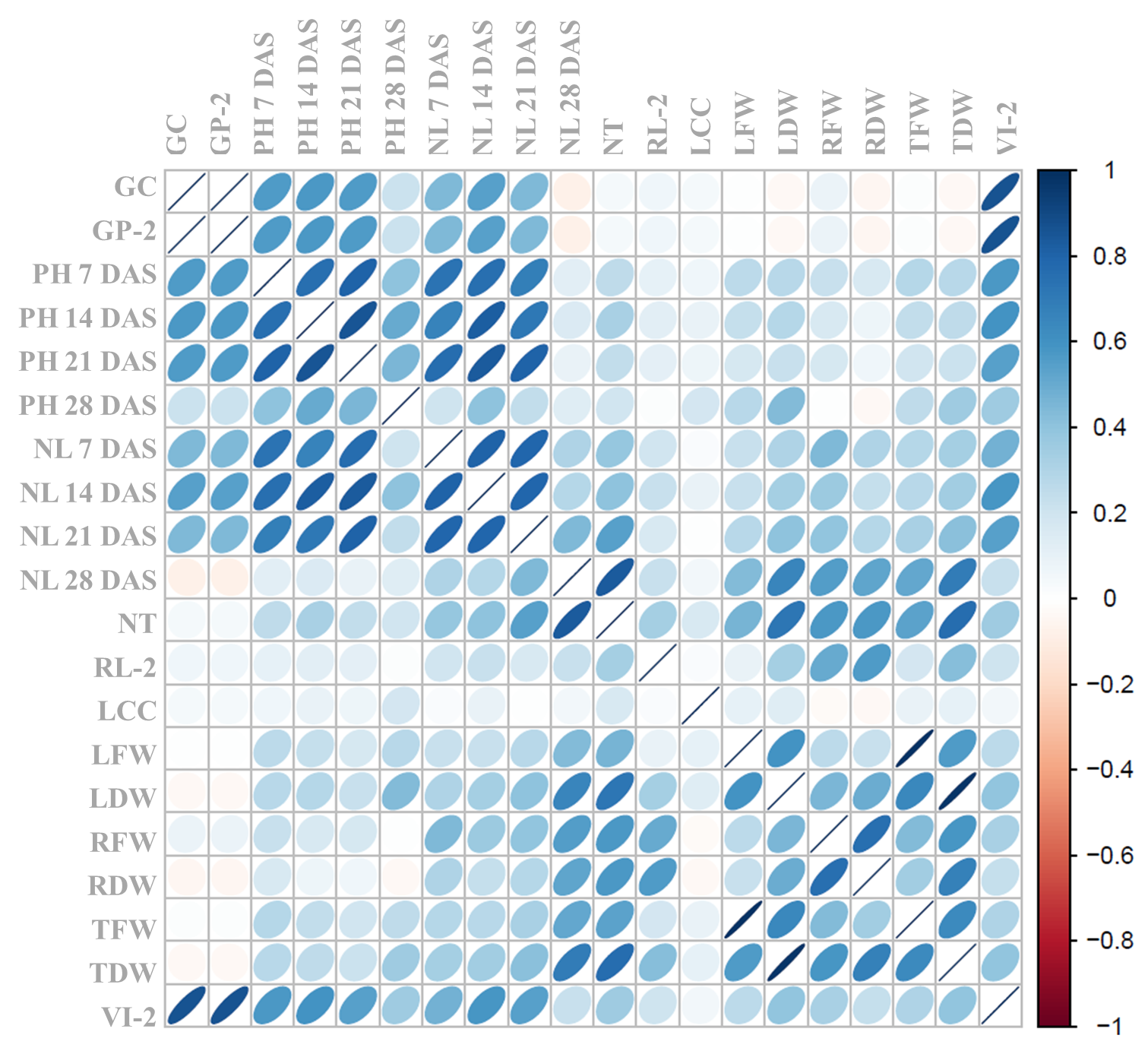
**

**Supplemental Fig. 2 Correlation matrix of early seedling vigor (ESV) related traits.** The scale represents the values of the Pearson coefficient between the ESV-related traits using the mean value of each trait. The color of bars (red, blue, and white) indicates the positive, negative, and no significant correlations between the traits, respectively. Abbreviations: DAS, days after sowing; GC, germination count; GP-2, germination percentage; PH, plant height at 7, 14, 21 and 28 DAS; NL, number of leaves at 7, 14, 21 and 28 DAS; NT, number of tillers; LCC, leaf chlorophyll content; LFW, leaf fresh weight; LDW, leaf dry weight; RFW, root fresh weight; RDW root dry weight; TFW, total fresh weight; TDW, total dry weight; VI-2, vigor index; WD, weed density; WFW, weed fresh weight; and WDW, weed dry weight.
