# Supplementary material for: Identification of quantitative trait loci governing early germination and seedling vigor traits related to weed competitive ability in rice
Source: Euphytica. 2020 Sep 19;216(10):159. doi: 10.1007/s10681-020-02694-8 (PMC7510932; doi:10.1007/s10681-020-02694-8)
Supplement: Supplementary file 2 — Supplementary material 2 (DOC 68 kb) [file 10681_2020_2694_MOESM2_ESM.doc]

**Supplementary Table 1** Descriptive statistics of all the traits measured for weed competitive ability (WCA) in rice.

|  | **Early seed germination (ESG) traits** | | **Min** | **Max** | **Mean** | **SD** | **CV (%)** | H^2^ |
| --- | --- | --- | --- | --- | --- | --- | --- | --- |
| 1 | 1st germination count | 1st GC | 0 | 25 | 9.51 | 5.87 | 44.05 | 0.49 |
| 2 | 2nd germination count | 2nd GC | 1 | 25 | 15.98 | 5.49 | 21.93 | 0.63 |
| 3 | Germination percentage | GP-1 | 2 | 100 | 63.77 | 22.25 | 22.43 | 0.64 |
| 4 | Shoot length (cm) | SL | 27.2 | 127.40 | 63.96 | 13.22 | 18.58 | 0.13 |
| 5 | Root length (cm) | RL-1 | 25.0 | 133.80 | 66.71 | 19.45 | 24.45 | 0.17 |
| 6 | Total fresh weight of germinated seeds (g) | TFGS | 0.12 | 1.26 | 0.67 | 0.18 | 19.42 | 0.28 |
| 7 | Total dry weight of germinated seeds (g) | TDGS | 0.01 | 0.57 | 0.29 | 0.08 | 17.83 | 0.48 |
| 8 | Average fresh weight (g) | AFW | 0.016 | 0.23 | 0.05 | 0.03 | 48.25 | 0.43 |
| 9 | Average dry weight (g) | ADW | 0.005 | 0.093 | 0.02 | 0.01 | 33.27 | 0.15 |
| 10 | Vigor index | VI-1 | 0.01 | 44 | 19.71 | 9.48 | 31.53 | 0.56 |
|  | **Early seedling vigor (ESV) traits** | | | | | | |  |
| 1 | Germination count | GC | 1 | 5 | 3.33 | 1.74 | 45.42 | 0.17 |
| 2 | Germination percentage | GP-2 | 10 | 100 | 66.53 | 34.89 | 45.42 | 0.19 |
| 3 | Seedling plant height (cm) at 7 DAS | PH at 7 DAS | 8.5 | 26.2 | 15.30 | 6.02 | 36.82 | 0.01 |
| 4 | Seedling plant height (cm) at 14 DAS | PH at 14 DAS | 20.3 | 60.2 | 26.87 | 9.70 | 30.89 | 0.06 |
| 5 | Seedling plant height (cm) at 21 DAS | PH at 21 DAS | 35.0 | 81.0 | 44.58 | 14.94 | 30.06 | 0.01 |
| 6 | Seedling plant height (cm) at 28 DAS | PH at 28 DAS | 53.7 | 87.2 | 68.81 | 13.42 | 15.03 | 0.04 |
| 7 | Number of leaves at 7 DAS | NL at 7 DAS | 2 | 3 | 2.03 | 0.80 | 34.21 | 0.05 |
| 8 | Number of leaves at 14 DAS | NL at 14 DAS | 2 | 6 | 3.82 | 1.38 | 31.09 | 0.04 |
| 9 | Number of leaves at 21 DAS | NL at 21 DAS | 5 | 16 | 8.61 | 3.31 | 32.40 | 0.18 |
| 10 | Number of leaves at 28 DAS | NL at 28 DAS | 8 | 35 | 14.04 | 4.50 | 25.75 | 0.04 |
| 11 | Number of tillers | NT | 2 | 7 | 3.46 | 1.04 | 22.25 | 0.01 |
| 12 | Leaf chlorophyll content | LCC | 32.5 | 154.8 | 36.14 | 9.38 | 23.25 | 0.35 |
| 13 | Root length (cm) | RL-2 | 8.8 | 69.1 | 14.35 | 5.23 | 30.67 | 0.03 |
| 14 | Leaf fresh weight (g) | LFW | 1.73 | 41.9 | 6.80 | 3.08 | 39.74 | 0.41 |
| 15 | Leaf dry weight (g) | LDW | 0.30 | 2.2 | 0.96 | 0.33 | 29.91 | 0.30 |
| 16 | Root fresh weight (g) | RFW | 0.19 | 3.6 | 0.91 | 0.56 | 45.26 | 0.02 |
| 17 | Root dry weight (g) | RDW | 0.05 | 0.5 | 0.16 | 0.09 | 40.52 | 0.02 |
| 18 | Total fresh weight (g) | TFW | 2.01 | 42.2 | 7.71 | 3.31 | 37.36 | 0.36 |
| 19 | Total dry weight (g) | TDW | 0.38 | 2.6 | 1.11 | 0.38 | 29.69 | 0.12 |
| 20 | Vigor index | VI-2 | 0 | 170.2 | 76.08 | 44.82 | 50.08 | 0.25 |

DAS, Days after sowing; SD, Standard deviation; CV, Coefficient of variation; H^2^, Broad sense heritability.
